# N-Acetylated Proline-Glycine-Proline Accelerates Cutaneous Wound Healing and Neovascularization by Human Endothelial Progenitor Cells

**DOI:** 10.1038/srep43057

**Published:** 2017-02-23

**Authors:** Yang Woo Kwon, Soon Chul Heo, Tae Wook Lee, Gyu Tae Park, Jung Won Yoon, Il Ho Jang, Seung-Chul Kim, Hyun-Chang Ko, Youngjae Ryu, Hyeona Kang, Chang Man Ha, Sang Chul Lee, Jae Ho Kim

**Affiliations:** 1 Department of Physiology, Pusan National University School of Medicine, Yangsan 50612, Republic of Korea; 2 Department of Oral Biochemistry and Molecular Biology, Pusan National University School of Dentistry, Yangsan 50612, Republic of Korea; 3 Department of Obstetrics and Gynecology, Pusan National University School of Medicine, Yangsan 50612, Republic of Korea; 4 Department of Dermatology, Pusan National University School of Medicine, Yangsan 50612, Republic of Korea; 5 Korea Brain Research Institute, Daegu 41068, Republic of Korea; 6 Functional Genomics Research Center, KRIBB, Daejeon 34141, Republic of Korea; 7 Research Institute of Convergence Biomedical Science and Technology, Pusan National University Yangsan Hospital, Yangsan 50612, Republic of Korea

## Abstract

Human endothelial progenitor cells (hEPCs) are promising therapeutic resources for wound repair through stimulating neovascularization. However, the hEPCs-based cell therapy has been hampered by poor engraftment of transplanted cells. In this study, we explored the effects of N-acetylated Proline-Glycine-Proline (Ac-PGP), a degradation product of collagen, on hEPC-mediated cutaneous wound healing and neovascularization. Treatment of hEPCs with Ac-PGP increased migration, proliferation, and tube-forming activity of hEPCs *in vitro*. Knockdown of CXCR2 expression in hEPCs abrogated the stimulatory effects of Ac-PGP on migration and tube formation. In a cutaneous wound healing model of rats and mice, topical application of Ac-PGP accelerated cutaneous wound healing with promotion of neovascularization. The positive effects of Ac-PGP on wound healing and neovascularization were blocked in CXCR2 knockout mice. In nude mice, the individual application of Ac-PGP treatment or hEPC injection accelerated wound healing by increasing neovascularization. Moreover, the combination of Ac-PGP treatment and hEPC injection further stimulated wound healing and neovascularization. Topical administration of Ac-PGP onto wound bed stimulated migration and engraftment of transplanted hEPCs into cutaneous dermal wounds. Therefore, these results suggest novel applications of Ac-PGP in promoting wound healing and augmenting the therapeutic efficacy of hEPCs.

Diabetic patients frequently suffer from impaired wound healing, with a lifetime risk of 15% for development of diabetic skin ulcerations^1^. Diabetic ulcers have a poor prognosis, and lead to surgical removal of bone in 15–27% of patients^2^. Therefore, development of therapeutics for chronic diabetic wounds is expected to significantly improve the impaired quality of life of people with chronic wounds. Cutaneous wound healing is a complex process involving the interaction among different cells in the injured tissues^3^. These cells contribute to wound healing process by stimulating inflammation, granulation tissue formation, angiogenesis, re-epithelialization, and remodeling^4^. Angiogenesis is defined as the formation of new capillaries from pre-existing blood vessels^5^. Formation of a new vasculature is essential for the removal of debris, providing nutrients and oxygen to the metabolically active wound bed. Therefore, angiogenesis is a critical step for wound healing processes and insufficient angiogenesis can result in impaired wound healing and chronic wound formation^6^. Angiogenic factors, including stromal cell-derived factor and vascular endothelial growth factor (VEGF), are part of angiogenesis^7^. Accumulating evidence suggests that chronic wound recovery is promoted by application of topical cytokines and growth factors^8^,^9^. However, expression and purification of these proteinous cytokines for therapeutic drug development is difficult. Instead, development of synthetic small peptides for stimulation of the wound healing processes will be useful for treatment of diabetic wounds.

Endothelial progenitor cells (EPCs) are a rare population of adult stem cells that contribute to postnatal vasculogenesis and angiogenesis after ischemic injury^10^,^11^. EPCs migrate from bone marrow to the circulation and to the site of tissue injury to augment neovascularization through direct incorporation or paracrine effect^12^,^13^. Neovascularization, by either vasculogenesis or angiogenesis, is essential for proper wound healing^14^. During typical wound healing, EPCs are recruited to the injury site and contribute to wound revascularization and timely healing. Impaired wound revascularization delays the healing process, and EPC numbers and activities in chronic wounds are decreased^15^,^16^,^17^. In EPC recruitment, breakdown and remodeling of extracellular matrix (ECM) component at the basement membrane of the vasculature and in the interstitial space accompany the invasion of EPCs to the injury site^18^,^19^. In addition to mobilization and recruitment of autologous EPCs, exogenous addition of EPCs is beneficial on the acceleration of wound healing^20^,^21^. Though EPCs hold promise as a powerful tool for treating wounds, the performance in clinical settings has not satisfied expectations, i.e. improvement in the therapeutic efficacy of EPC treatment^22^.

N-acetylated proline-glycine-proline (Ac-PGP) is a tripeptide derived from collagen after multiple proteolysis steps including metalloprotease (MMP) 8, MMP9, and prolyl endopeptidase^23^. Ac-PGP is a strong chemokine for neutrophils, which is mediated through chemokine C-X-C motif receptor 1 (CXCR1) and CXCR2^24^. Ac-PGP is important in neutrophil influx in chronic inflammatory diseases such as chronic obstructive pulmonary disease, cystic fibrosis, and inflammatory bowel disease^25^,^26^,^27^,^28^. Collagen is a major molecule found in healing wounds and is critical for wound revascularization^29^. In tissue injury with acute wound healing, the ingrowth of granulation tissue with new blood vessels occurs at day 5, wherein the proteases, such as matrix metalloproteinases and collagenases, are critical for the ingrowth into the wound^30^. In the proliferation stage of wound healing, collagen deposition and angiogenesis proceed concurrently, and MMPs promote angiogenesis via breakdown of the ECM including collagen^31^. However, the effect of collagen-derivative Ac-PGP on neovascularization or wound healing has not been studied. Moreover, it has not been explored whether Ac-PGP can improve the EPCs-stimulated wound healing and neovascularization.

The present study investigated the effects of Ac-PGP on hEPC-mediated cutaneous wound healing *in vivo*. In addition, the effects of Ac-PGP on migration and angiogenic capacities of hEPCs and the role of CXCR2 in the Ac-PGP-induced cellular responses were explored.

## Results

### Ac-PGP stimulates migration, tube formation, and proliferation of hEPCs

**Figure 1:**
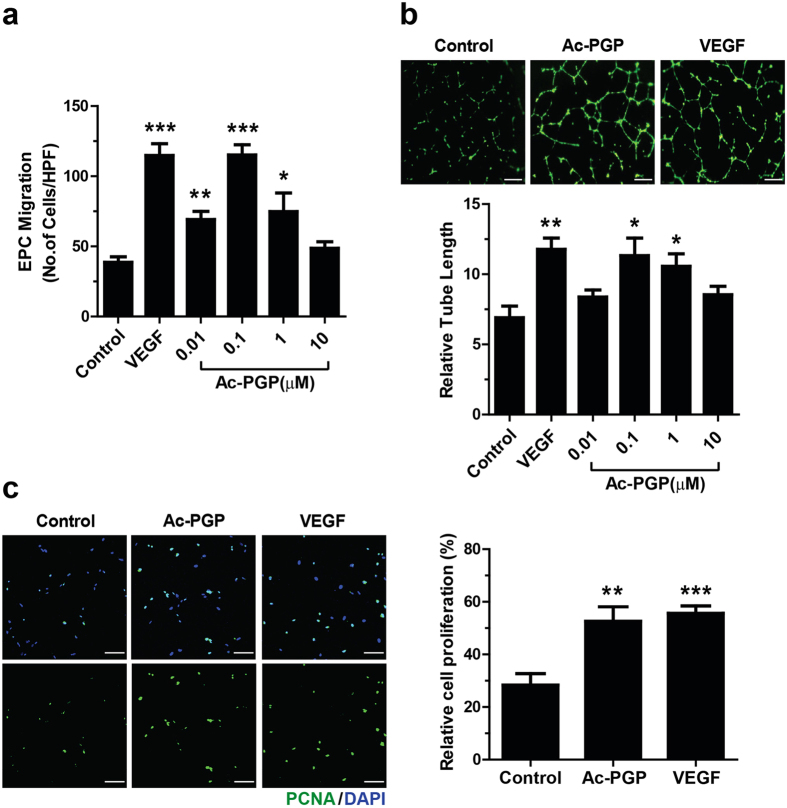
Effects of Ac-PGP on the migration, tube formation, and proliferation activities of hEPCs. (**a**) Migration of hEPCs was measured using a chemotaxis chamber in response to VEGF (10 ng/ml) or various concentrations of Ac-PGP after 12 hour incubation. Data indicate mean ± SD. *p < 0.05, **p < 0.01, ***p < 0.001 versus control (n = 6). (**b**) Representative images of tube formation in response to Ac-PGP (0.1 μM) or VEGF (10 ng/ml) are shown (upper panels). Tube formation was quantified by measuring the length of tubes (lower panel). Data indicate mean ± SD. *p < 0.05, **p < 0.01, versus control (n = 6). Bar = 50 μm. (**c**) The proliferative effect of Ac-PGP on hEPCs, hEPCs was measured by staining with anti-PCNA antibody (green). Nuclei were counterstained with DAPI (blue). Numbers of PCNA-positive nuclei per field were counted and expressed as the relative percentage of total cells. Data indicate mean ± SD. **p < 0.01, ***p < 0.001 versus control (n = 6). Bar = 100 μm.

During the wound healing process, EPCs migrate toward the wound site and contribute to wound repair by promoting neovascularization^32^. To evaluate if Ac-PGP induces chemotaxis of human EPCs (hEPCs), we tested the effect of Ac-PGP on the migration capacity of hEPCs with the chamber migration assay. As shown in Fig. 1a, Ac-PGP promoted the migration of hEPCs with maximal effect at 0.1 μM to the level comparable to the migration by VEGF. When Ac-PGP was added to the tube formation assay of hEPCs, 0.1 and 1 μM of Ac-PGP significantly increased the tube-forming activity of hEPCs as potent as VEGF (Fig. 1b). In addition, when the effect of Ac-PGP on the proliferation of hEPCs was evaluated by measuring PCNA expression, the incubation of hEPCs with 0.1 μM Ac-PGP promoted the proliferation of hEPCs to the level comparable to VEGF (Fig. 1c). When the dose dependency of Ac-PGP on proliferation of hEPCs was evaluated, increasing dose of Ac-PGP showed a biphasic effect, as in migration and tube formation assays, without showing cellular toxicity (Supplementary Fig. S1). These results suggest that Ac-PGP is a novel angiogenic peptide, which stimulates migration, tube formation, and proliferation of hEPCs.

### CXCR2 is required for Ac-PGP-induced stimulation of migration and tube formation in hEPCs

**Figure 2:**
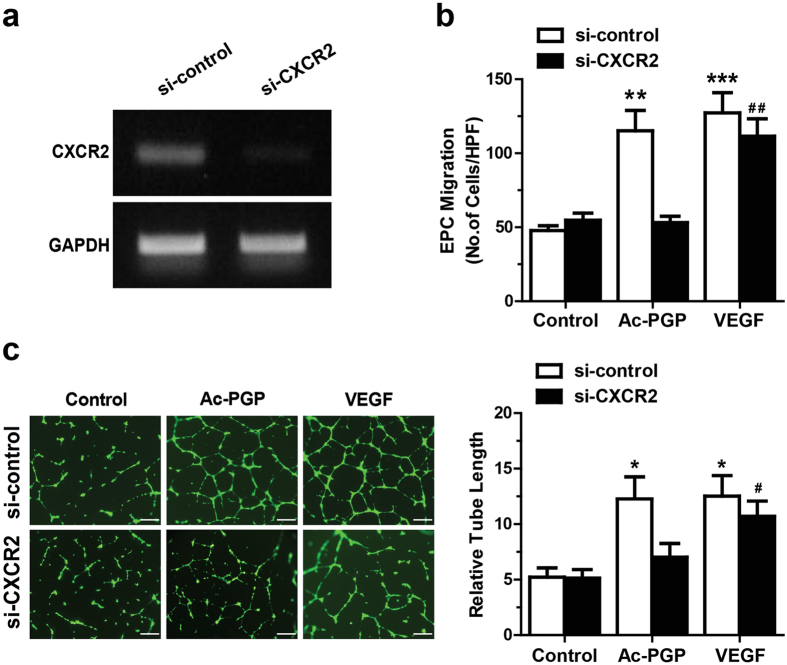
Role of CXCR2 in the Ac-PGP-induced migration and tube formation of hEPCs. (**a**) hEPCs were transfected with control siRNA (si-control) or CXCR2 specific siRNA (si-CXCR2), and mRNA levels of CXCR2 and GAPDH were determined by reverse transcription-polymerase chain reaction. (**b**) Silencing of CXCR2 abrogates Ac-PGP (0.1 μM)-stimulated migration of hEPCs but not VEGF-stimulated migration. Data indicate mean ± SD. **p < 0.01, ***p < 0.001 versus control, ^##^p < 0.01, si-CXCR2 VEGF versus si-CXCR2 control (n = 8) (**c**) Representative images of tube formation by hEPCs in response to Ac-PGP (0.1 μM) or VEGF (10 ng/ml) with or without CXCR2 knockdown are shown (left panels). Quantitative analysis of tube length is shown in the right panel. Data indicate mean ± SD. *p < 0.5, versus control, ^#^p < 0.05, si-CXCR2 VEGF versus si-CXCR2 control (n = 4). Bar = 50 μm.

CXCR2 is a receptor for Ac-PGP^24^, and hEPCs showed the high expression of CXCR2 (Supplementary Fig. S2). To investigate the involvement of CXCR2 in Ac-PGP-induced stimulation of angiogenic capacities of hEPCs, we evaluated the effects of small interfering RNA (siRNA)-mediated knockdown of CXCR2 in the angiogenic activities of hEPCs. When hEPCs were transfected with CXCR2-targetting siRNA (si-CXCR2), CXCR2 expression decreased significantly at mRNA level (Fig. 2a). CXCR2 knockdown significantly decreased Ac-PGP-induced migration of hEPCs, while VEGF-induced migration was not affected (Fig. 2b). When hEPCs were subjected to the tube formation assay with or without CXCR2 knockdown, CXCR2 knockdown significantly decreased tube-forming abilities of hEPCs stimulated by Ac-PGP but not VEGF (Fig. 2c). To verify the importance of CXCR2 in mediating Ac-PGP-induced cellular responses, hEPCs were pretreated with SB225002, a CXCR2 antagonist, and subjected to migration and tube formation assays. Blockade of CXCR2 by SB225002 significantly reduced Ac-PGP-induced migration and tube formation of hEPCs (Supplementary Fig. S3). These results suggest that CXCR2 is crucial in the Ac-PGP-induced migration and tube formation of hEPCs.

### Ac-PGP treatment accelerates cutaneous wound healing in rats

**Figure 3:**
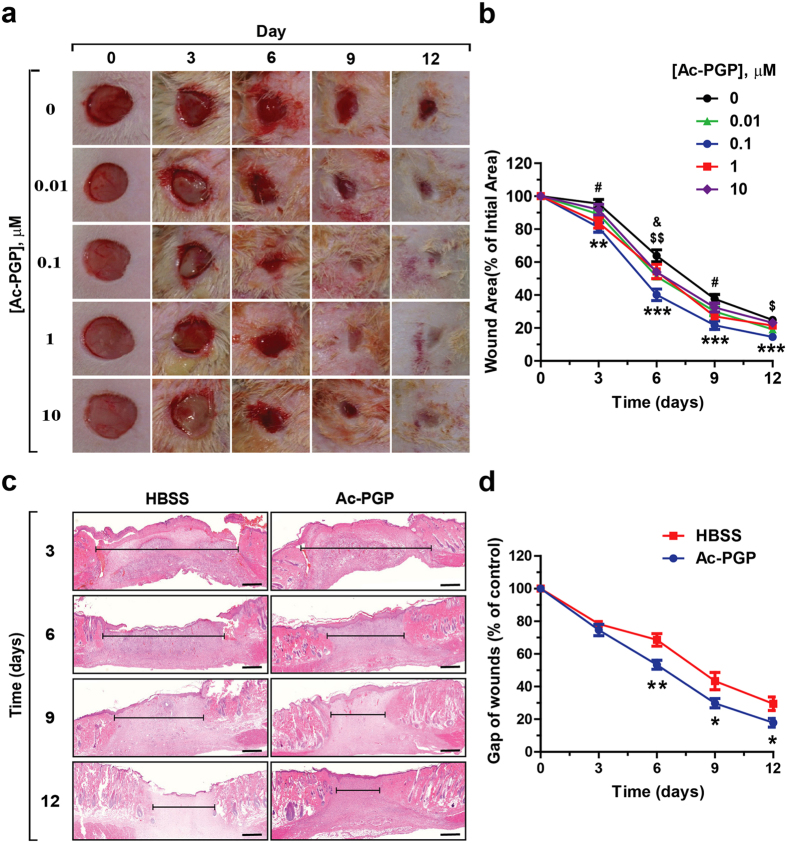
Effects of Ac-PGP treatment on the cutaneous wound healing. (**a**) Excisional skin wounds introduced by 8-mm biopsy punch on the dermal skin in rats were treated daily with HBSS or indicated concentration of Ac-PGP. Representative images taken at indicated days after introducing wounds are shown. (**b**) Quantitative analysis of wound area at the indicated days in comparison with the original wound using Image J software (version 1.50i) is shown. Data indicate mean ± SD. ^$^p < 0.05, ^$$^p < 0.01 0.01 uM Ac-PGP versus 0 uM Ac-PGP; **p < 0.01, ***p < 0.001 0.1 uM Ac-PGP versus 0 uM Ac-PGP; ^#^p < 0.05 1 uM Ac-PGP versus 0 uM Ac-PGP; ^&^p < 0.05 10 uM Ac-PGP versus 0 uM Ac-PGP (n = 12). (**c**) Representative images of cutaneous wound tissues at the indicated days after H&E staining are shown. Wound gap, the distance between the advancing edges of wounds, is marked by a black line. Bar = 200 μm. (**d**) Quantitative analysis of the wound gap on the indicated days during recovery the period is shown. The wound gap was measured using the Image J software program (version 1.50i). Data indicate mean ± SD. *p < 0.05, **p < 0.01 versus HBSS (n = 6).

To evaluate the effect of Ac-PGP on cutaneous wound healing, we applied Ac-PGP to the skin wounds in Sprague–Dawley rats. After creating an 8 mm excisional wound in the dorsal area, varying concentrations of Ac-PGP were applied daily for 12 days. As shown in Fig. 3a, Ac-PGP treatment accelerated wound healing. Wound areas showed a time dependent decrease under all experimental condition with a maximum effect at 0.1 μM on day 6. When wound diameters were measured, the wound closure rate in Ac-PGP-treated group was accelerated by 11 to 23% in comparison with Hanks’ Balanced Salt solution (HBSS)-treated control group (Fig. 3b). Histological analysis of the cross section by H&E staining showed that the wound gap, the distance of between the advancing edges of epithelial layers, decreased by Ac-PGP treatment in comparison with HBSS treatment control, indicating the promotion of re-epithelialization (Fig. 3c,d). These results suggest that Ac-PGP treatment accelerates cutaneous wound healing in rat dermal wound model.

### Ac-PGP treatment promotes neovascularization and infiltration of monocytes/macrophages in cutaneous wounds

**Figure 4:**
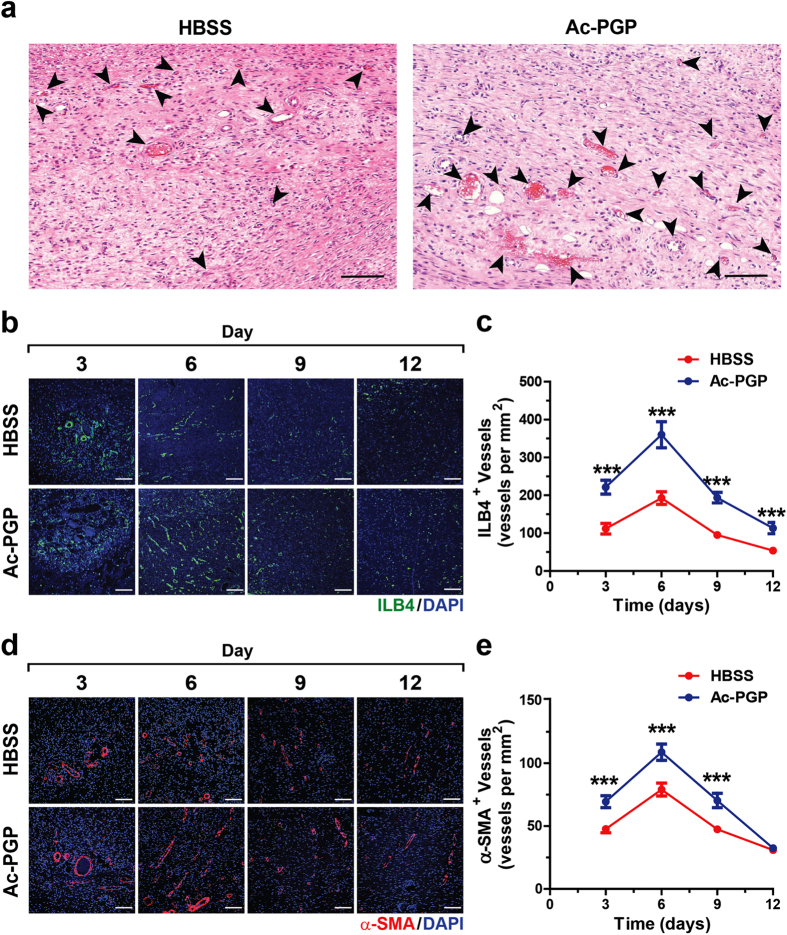
Effect of Ac-PGP treatment on neovascularization during the cutaneous wound healing. (**a**) Representative H&E staining images of cutaneous wound in rats on day 6 after treatment of HBBS or Ac-PGP (0.1 μM) are shown. Functional blood vessels that include red blood cells are indicated by arrows. Bar = 100 μm. (**b**) Fluorescence images of ILB4 staining (green) in the rat dermal area at indicated days after introducing wound followed by treatment with HBSS or Ac-PGP (0.1 μM) are shown. Nuclei were counterstained with DAPI (blue). Bar = 100 μm. (**c**) Quantitative analysis of ILB4-positive blood vessels in the dermal area at indicated days after introducing wound is shown. Data indicate mean ± SD. *** < 0.001 versus HBSS (n = 8). (**d**) Fluorescence images of α-SMA staining (red) in the rat dermal area at indicated days after introducing wound followed by treatment with HBSS or Ac-PGP (0.1 μM) are shown. Nuclei were counterstained with DAPI (blue). Bar = 100 μm. (**e**) Quantitative analysis of α-SMA-positive vessels in the dermal area at indicated days after introducing wound is shown. Data indicate mean ± SD. *** < 0.001 versus HBSS (n = 8).

During cutaneous wound healing, neovascularization is of primary importance for the repair and recovery of skin tissue^33^. To evaluate whether Ac-PGP treatment promotes neovascularization in cutaneous wound healing, we analyzed the number of blood vessels in rat dermal wounds with or without Ac-PGP treatment after creation of an excisional wound in the dorsal area. Histological analysis of the cross section of day 6 dermal wound by H&E staining showed that Ac-PGP treatment increased the number of blood vessels in the dermal area in comparison with HBSS treatment (Fig. 4a). When dermal samples of day 3, 6, 9, and 12 after creation of the excisional wound with or without Ac-PGP treatment were stained with isolectin B4 (ILB4), which specifically labels blood vessels, the Ac-PGP-treated dermal area had more blood vessels than wounds with HBSS treatment, with a maximum increase at day 6 (Fig. 4b,c). In addition, the increase in the number of α-smooth muscle actin (α-SMA)-positive mature vessels was evident in Ac-PGP-treated group in comparison with HBSS-treated control group on day 3, day 6, and day 9 during the recovery (Fig. 4d,e). Immune cell infiltration is a contributing factor at early phase of cutaneous wound healing^34^. When monocyte/macrophage infiltration was evaluated, Ac-PGP treatment significantly increased the infiltration of CD68-positive cells at day 3 but not afterward (Supplementary Fig. S4). Taken together, these results suggest that Ac-PGP treatment during cutaneous wound healing promotes neovascularization and infiltration of monocytes/macrophages.

### CXCR2 is required for Ac-PGP-induced promotion of wound healing, neovascularization, and infiltration of monocytes/macrophages

**Figure 5:**
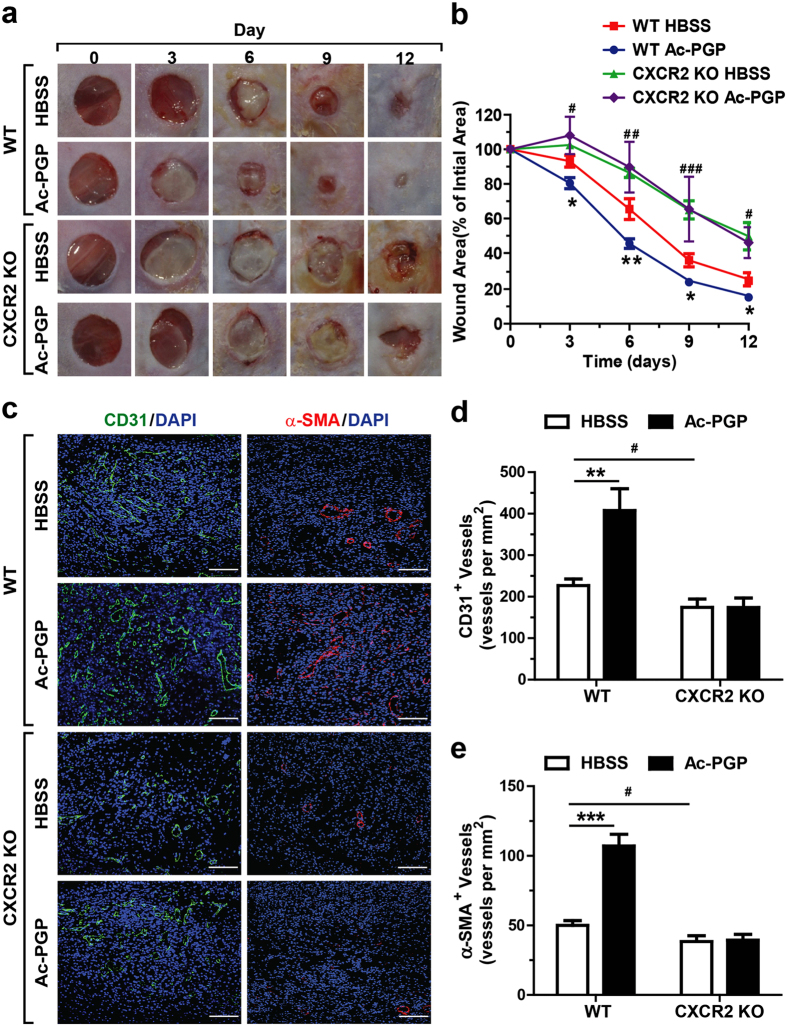
Effects of CXCR2 on the Ac-PGP–stimulated acceleration of wound healing and neovascularization. (**a**) Representative images of wounds at indicated days with HBBS or Ac-PGP (0.1 μM) treatment after creating excisional skin wounds by 6-mm biopsy punch in wild-type (WT) or CXCR2-knockout (CXCR2 KO) mice are shown. (**b**) Quantitative analysis of the wound area at indicated days in comparison with the original wound during the recovery period is shown. The wound area was measured by using Image J software program (version 1.50i). Data indicate mean ± SD. *p < 0.05, **p < 0.01 WT Ac-PGP versus WT HBSS; ^#^p < 0.05, ^##^p < 0.01, ^###^p < 0.001 WT HBSS versus CXCR2 KO HBSS (n = 12–20). (**c**) Representative images of mouse skin wound samples at day 6 after immunostaining with anti-CD31 antibody (green) or anti-α-SMA antibody (red) are shown. Nuclei were counterstained with DAPI (blue). Bar = 100 um. (**d**) Quantitative analysis of CD31-positive blood vessels in the dermal area of day 6 skin wound samples is shown. Data indicate mean ± SD. **p < 0.01 WT Ac-PGP versus WT HBSS, ^#^p < 0.05 WT HBSS versus CXCR2 KO HBSS (n = 8). (**e**) Quantitative analysis of α-SMA-positive vessels in the dermal area of day 6 skin wound samples is shown. Data indicate mean ± SD. ***p < 0.01 WT Ac-PGP versus WT HBSS, ^#^p < 0.05 WT HBSS versus CXCR2 KO HBSS (n = 8).

To evaluate the role of CXCR2 in Ac-PGP-induced promotion of cutaneous wound healing, we compared the effect of topical Ac-PGP treatment in wild type mice and CXCR2 knockout mice. After making an excisional wound on the dorsal area, Ac-PGP (0.1 μM) or HBSS were applied daily to the wound area with examinations at every three days for 12 days. As shown in Fig. 5a,b, Ac-PGP treatment significantly accelerated the cutaneous wound healing. However, the stimulatory effect of Ac-PGP on wound healing was completely abrogated in CXCR2 knockout mice. When day 6 skin samples were subjected to immunostaining with anti-CD31 or anti-α-SMA antibodies, Ac-PGP treatment in wild type mice significantly increased the number of CD31-positive vessels in the dermal area in comparison with HBSS treatment (Fig. 5c,d). However, Ac-PGP treatment in CXCR2 knockout mice showed little change in the number of CD31-positive vessels in the dermal area. Similarly, Ac-PGP treatment increase d α-SMA-positive mature vessels in wild type mice but not in CXCR2 knockout mice (Fig. 5c,e). In addition, Ac-PGP-induced CD68-positive monocyte/macrophage infiltration was not observed in CXCR2 knockout mice (Supplementary Fig. S5). These results suggest that CXCR2 is responsible for Ac-PGP-induced acceleration of wound healing, promotion of neovascularization, and infiltration of monocytes/macrophages.

### Ac-PGP treatment augments hEPC-induced promotion of wound healing and neovascularization

**Figure 6:**
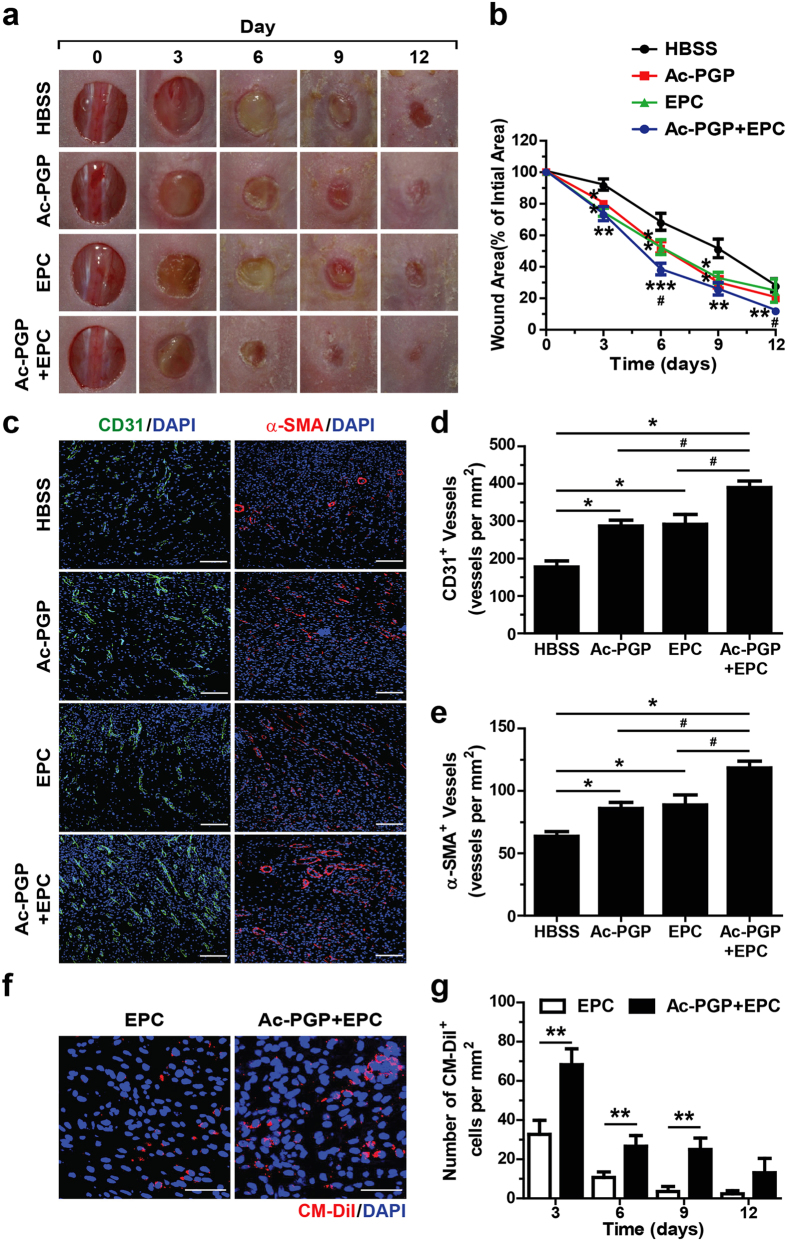
Effects of combined treatment of Ac-PGP and hEPC on the cutaneous wound healing and neovascularization. (**a**) Excisional skin wounds were introduced by 8-mm biopsy punch on the dermal skin of BALB/CA-nu/nu mice, followed by treatment with HBSS, Ac-PGP (0.1 μM), CM-DiI labeled-hEPC injection, and the combination of Ac-PGP (0.1 μM) and hEPCs injection. Representative images at indicated days during the recovery period are shown. (**b**) Quantitative analysis of the wound area at indicated days in comparison with the original wound during the recovery period is shown. The wound area was measured by using Image J software program (version 1.50i). Data indicate mean ± SD. *p < 0.05, **p < 0.01, *** < 0.001 versus HBSS, ^#^p < 0.05 Ac-PGP + hEPC versus Ac-PGP or hEPCs (n = 8). (**c**) Representative images of mouse skin wound samples at day 6 after immunostaining with anti-CD31 (green) or α-SMA (red) antibodies are shown. Nuclei were counterstained with DAPI (blue). Bar = 100 μm. (**d**) Quantitative analysis of CD31-positive blood vessels in the dermal area of wound is shown. *p < 0.05 versus HBSS, ^#^p < 0.05 Ac-PGP + hEPCs versus Ac-PGP or hEPCs (n = 8). (**e**) Quantitative analysis of α-SMA-positive vessels in the dermal area of wound is shown. *p < 0.05 versus HBSS, ^#^p < 0.05 Ac-PGP + hEPCs versus Ac-PGP or hEPCs (n = 8). (**f**) Representative images of day 6 mouse skin wound samples injected with CM-DiI-labeled hEPCs with or without the combination of Ac-PGP treatment (0.1 μM) are shown. Nuclei were counterstained with DAPI (blue). Bar = 50 μm. (**g**) Quantitative analysis of CM-DiI-positive hEPCs in dermal area of day 3, 6, 9, and 12 skin wound samples is shown. Data indicate mean ± SD. ** < 0.01 Ac-PGP + hEPC versus hEPCs (n = 6).

To evaluate if co-treatment with Ac-PGP and hEPCs can further increase the therapeutic efficacy of hEPCs in cutaneous wound healing, we compared the individual application of Ac-PGP or hEPCs with the combination treatment of Ac-PGP and hEPCs in nude mice. After creating an excisional wound in the dorsal area, CM-DiI-labeled hEPCs were injected to four peripheral sites adjacent to the wound at day 0 and Ac-PGP was topically applied to cutaneous wounds daily. As shown in Fig. 6a,b, both hEPC injection and Ac-PGP treatment accelerated cutaneous wound healing with a similar efficacy. The combination of hEPC injection and Ac-PGP treatment significantly accelerated wound healing more than control groups treated with either hEPCs or Ac-PGP, suggesting that Ac-PGP treatment augments the therapeutic efficacy of hEPCs. When skin wound samples were subjected to immunostaining wound with anti-CD31 or anti-α-SMA antibodies, the individual application of Ac-PGP treatment or hEPC injection increased CD31-positive blood vessels (Fig. 6c,d) and α-SMA-positive mature vessels (Fig. 6c,e) in the dermal area on day 6 in comparison with HBSS treatment control. Interestingly, the combination of Ac-PGP treatment and hEPC injection further increased CD31-positive vessels and α-SMA-positive mature vessels in the dermal area. We next examined whether Ac-PGP treatment had a positive effect on the survival of injected hEPCs. When the numbers of hEPCs in wounds were analyzed at day 3, 6, 9, and 12, Ac-PGP co-treatment significantly increased the numbers of CM-DiI-positive hEPCs within the wound area at day 3, 6, and 9 in comparison with hEPC injection alone (Fig. 6f,g), suggesting enhanced engraftment of transplanted hEPCs into wound bed in the presence of Ac-PGP. These results suggest that combination treatment of hEPCs and Ac-PGP accelerates neovascularization and wound repair by promoting the survival of transplanted hEPCs.

To further verify the concerted action of Ac-PGP treatment and hEPC injection on neovascularization during wound healing process, we subjected the skin wound samples to CUBIC tissue clearing protocol followed by whole mount immunostaining with anti-CD31 antibody (Supplementary Fig. S6a)^35^. When whole mount samples corresponding to a quarter of the wound area were analyzed by light sheet microscope and 3D image reconstruction, the individual treatment of Ac-PGP or hEPC injection showed the increase of CD31-positive blood vessels in the dermal area in comparison with HBSS treatment (Supplementary Fig. S6b, Supplementary Video S1,S2,S3,S4). Moreover, the combination of Ac-PGP treatment and hEPC injection further increased the numbers of CD31-positive blood vessels in the dermal area in comparison with individual treatments. These results strongly support the conclusion that the combination of Ac-PGP treatment along with hEPC injection further enhances the stimulatory effects of hEPCs on neovascularization and wound repair.

## Discussion

Ac-PGP is a degradation product from collagen involving multistep proteolysis^23^. In the present study, we demonstrated that Ac-PGP stimulated migration, proliferation, and tube-forming ability of hEPCs *in vitro* and enhanced neovascularization *in vivo*. The effects of Ac-PGP on cellular responses of hEPCs were biphasic with increasing dose of Ac-PGP. The biphasic effect could be contributed by the presence of negative feedback or cellular toxicity. However, Ac-PGP did not show toxicity to hEPCs in all doses tested *in vitro*. Furthermore, topical application of the collagen-derived tripeptide Ac-PGP accelerated cutaneous wound healing and neovascularization. The beneficial effect of Ac-PGP on wound healing and neovascularization was blocked in CXCR2 knockout mice. Moreover, knockdown or chemical blockade of CXCR2 significantly decreased Ac-PGP-induced migration and tube-forming ability of hEPCs. These results suggest that CXCR2 is crucial in Ac-PGP-mediated wound repair and angiogenesis. Although the stimulatory effect of Ac-PGP on wound healing and angiogenesis has not been reported, the involvement of CXCR2 in wound healing and angiogenesis has been well documented^36^. In CXCR2 knockout mice, a significant delay in neovascularization and impaired wound healing has been reported^37^. In healing human skin from excision wounds, IL-8, a strong agonist of CXCR2, is highly expressed in a band-like pattern immediately below the denuded wound surface and recruits neutrophils^38^. Although topical application of IL-8 in mouse wound healing mildly improved re-epithelialization, IL-8 expression peaks at day 1 and a prolonged exposure to IL-8 raises a concern for the delay in the resolution of pro-inflammatory responses^39^,^40^. These reports support the present study demonstrating that Ac-PGP accelerates cutaneous wound healing in a CXCR2-dependent mechanism, which suggest a novel application of Ac-PGP in wound repair.

hEPC injection to the peripheral sites of the wound accelerated wound healing, and co-treatment of Ac-PGP together with hEPC injection further boosted the wound healing process. Low numbers of EPCs or EPC dysfunction are associated with delayed or impaired wound healing with vascular insufficiency^41^,^42^,^43^. In experimental settings, hEPC treatment in wound healing models leads to positive outcome in wound healing^20^,^44^. In clinical settings, however, the efficacy of hEPC treatment for therapeutic angiogenesis has been controversial, which necessitates the development of a means to enhance the therapeutic effect of hEPCs^19^,^22^. In recent report, fibrin fragment E exhibited the higher effect on hEPC in adhesion, proliferation, and differentiation than fibrin, and co-administration of hEPCs with fibrin fragment E-enriched scaffold accelerated wound healing^45^. Collagen has been the material of choice for wound healing with applications in dressings, injectables, membranes, and skin grafts due to wide distribution, abundance, biocompatibility, and biodegradability^46^. Development of peptide-based products may reduce production cost with increased shelf life and concerns of potential disease transmission by using animal products^47^. A combination of hEPCs and Ac-PGP-based products may significantly increase the therapeutic effect of hEPCs in clinical trials.

Ac-PGP is a pro-inflammatory matrix-derived chemokine (matrikine) involved in the progression of chronic inflammatory diseases such as obstructive pulmonary disease, cystic fibrosis, and inflammatory bowel disease^25^,^26^,^27^. Ac-PGP stimulates polymorphonuclear cells including neutrophils, and the neutralization of Ac-PGP activity has been suggested as a therapeutic strategy. A complementary peptide to Ac-PGP, arginine-threonine-arginine (RTR), blocked Ac-PGP-induced emphysema and cigarette smoke-induced neutrophil infiltration^28^,^48^. The beneficiary effects of Ac-PGP, however, were suggested if Ac-PGP activity could be limited to cartilage endplate stem cell migration in intervertebral disc degeneration model in which the prolonged exposure to Ac-PGP converts cartilage endplate stem cells to a pro-inflammatory phenotype^49^. In experimental sepsis models, Ac-PGP treatment is therapeutically effective with increased IFN-γ production and decreased pro-inflammatory cytokine release^50^. In the present study, we showed that topical application of Ac-PGP accelerated wound healing along with promotion of neovascularization and infiltration of CD68-positive monocytes/macrophages. Moreover, combined treatment of Ac-PGP along with hEPCs potentiated the hEPC-mediated wound healing and neovascularization by enhancing the engraftment of transplanted hEPCs.

In the current study, we demonstrated the medicinal effect of collagen-derived tripeptide Ac-PGP on wound healing. Ac-PGP treatment accelerated wound healing with enhanced neovascularization, and the combination of hEPCs and Ac-PGP was the most effective. These results may contribute to the development of therapeutic products for acute and chronic wounds.

## Materials and Methods

### Materials

Recombinant human VEGF was purchased from R&D Systems (Minneapolis, MN, www.rndsystems.com). Anti-CD31 antibody (MEC 13.3) and growth factor reduced Matrigel were purchased from BD Biosciences (Bedford, MA). Anti-PCNA antibody (FL-261) was purchased from Santa Cruz Biotechnology, Inc. (Dallas, TX). Rabbit anti-α-SMA antibody (ab5694) was purchased from Abcam (Cambridge, United Kingdom). Biotinylated-ILB4 was purchased from Vector Laboratories (Burlingame, CA). Alexa 488 Streptavidin, Alexa 488 goat anti-rabbit, Alexa 488, 568 and Alexa 647 goat anti-rat secondary antibodies were purchased from Life Technologies (Carlsbad, CA). Ac-PGP (Ac-Pro-Gly-Pro-OH, purity >95%) was purchased from Anaspec Inc. (Fremont, CA).

### Cell Culture

hEPCs were isolated from human umbilical cord blood and collected in disposable sterile pyrogen-free bags containing anticoagulant (Green Cross, Seoul, Korea). Written informed consent was obtained from all donors and the protocol was approved by the Institutional Review Board of Pusan National University Hospital (Permit Number: H-1302-005-015). The methods were carried out in accordance with the approved guidelines. Mononuclear cells (MNCs) were isolated from blood using Ficoll-Paque PLUS (17-1440-02, GE healthcare) as described previously. Cells were seeded on culture dishes coated with 0.1% gelatin and maintained in EGM-2MV BulletKit that is endothelial cell basal medium-2 (EBM-2) (Clonetics, San Diego, CA) supplemented with EGM-2 MV SingleQuots containing 5% fetal bovine serum (FBS), human VEGF, human fibroblast growth factor-2, human epidermal growth factor, insulin-like growth factor-1, and ascorbic acid. The medium was changed 24 hours after the initial plating for removal of non-adherent cells and was daily changed for the first week. Colonies of hEPCs appeared seven to ten days after the initial isolation. Non-adherent cells were removed, and the adherent cells were trypsinized and replated at a density of 1 × 10^6^ cells per well. Expression of hEPC-specific cell surface markers, including CD31, CD34, CD133, c-kit, Flk1, CXCR4, and CD144, was confirmed by flow cytometry analysis. Hematopoietic lineage markers such as CD11b, CD14, and CD45 were not expressed in hEPCs, indicating the commitment of hEPCs to the endothelial lineage. hEPCs were maintained at 37 °C in a 5% CO_2_ atmosphere.

### Cell migration

hEPC migration was assayed using a disposable 96-well chemotaxis chamber (Neuro Probe, Inc., Gaithersburg, MD). hEPCs were harvested with 0.05% trypsin containing 0.02% EDTA, washed once, and suspended in EBM-2 at a concentration of 1 × 10^4^ cells/ml. A membrane filter with 8-μm pores of the chemotaxis chamber was pre-coated overnight with 20 μg/ml rat-tail collagen at 4 °C, an aliquot (50 μL) of hEPC suspension was loaded into the upper chamber, and Ac-PGP or VEGF was then placed in the lower chamber. To investigate the involvement of CXCR2, hEPCs were pre-incubated with 10 μM SB225002 for 30 min before addition of Ac-PGP. After incubation of the cells for 12 h at 37 °C, 5% CO_2_ the filters were disassembled, and the upper surface of each filter was scraped free of cells by wiping it with a cotton swab. The number of cells that migrated to the lower surface of each filter was determined by counting the cells in four random locations under microscope at ×100 magnification after staining with Hoechst 33342.

### Tube formation

For tube formation of hEPCs, growth factor reduced Matrigel were added to 96-well culture plates and polymerized for 30 min at 37 °C. 1 × 10^4^ hEPCs were seeded on Matrigel coated plates and cultured in EBM-2 medium supplemented with 0.5% FBS, followed by treatment with Ac-PGP or VEGF. After incubation of the cells at 37 °C, 5% CO_2_ for 12 hours, the capillary structures were photographed with a digital camera in four random microscopic fields and quantified by measuring the capillary length using the Image J software (version 1.50i).

### Cell proliferation

The effects of Ac-PGP on hEPCs proliferation were investigated by immunocytochemistry. Cells were seeded on in with 0.1% gelatin coated 24-well culture plates and incubated with EBM-2 containing 0.5% FBS or supplemented 0.1 μM Ac-PGP or VEGF for 24 hours. hEPCs were fixed in phosphate-buffered saline (PBS) containing 4% paraformaldehyde for 15 min, permeabilized with PBS containing 0.2% Triton X-100 for 10 min, and blocked with PBS containing 5% bovine serum albumin. Specimens were incubated with rabbit anti-Proliferating Cell Nuclear Antigen (PCNA) antibody (Santa Cruz Biotechnology, Inc.) for two hours and Alexa 488 goat anti-rabbit secondary antibodies (Life Technologies, Carlsbad, CA) for one hour. The specimens were finally washed and mounted in Vectashield medium (Vector Laboratories, Burlingame, CA) with 40, 6-diamidino-2-phenylindole (DAPI), and images of the specimen were collected with laser scanning confocal microscope.

### Cellular toxicity

Apoptosis of hEPCs was investigated by terminal deoxynucleotidyl transferase-mediated dUTP nick endlabeling (TUNEL) assay. hEPCs were seeded on in with 0.1% gelatin coated 24-well culture plates and incubated with EBM-2 containing 0.1% FBS with or without VEGF or various concentrations of Ac-PGP for 24 hours. TUNEL assay was conducted according to the ApoAlert DNA Fragmentation Assay kit manual (Clontech Laboratories, Inc., Mountain View, CA). The apoptotic cells were quantified as the percentage of TUNEL-positive nuclei among total DAPI stained cells.

### Transfection with small interfering RNA and reverse transcription-polymerase chain reaction

The CXCR2 expression in hEPCs was silenced by transfection with small interfering RNA (siRNA) specific for CXCR2. CXCR2 mRNA level in hEPCs was determined by reverse transcription-polymerase chain reaction (RT-PCR) analysis. For siRNA experiments, hEPCs were seeded on 60-mm diameter dishes at 70% confluence, and transfected with control siRNA or siRNA specific for CXCR2 using Lipofectamine 2000 reagent according to the manufacturer’s instructions. Lipofectamine 2000 reagent was incubated with serum-free medium for 15 min, and respective siRNAs were added to the mixtures. After incubation for 15 min at room temperature, the mixtures were diluted with serum free medium and added to each well. The final concentration of siRNAs in each well was 10^−7^ M. After incubation of hEPCs with serum-free medium containing siRNAs for 4 h, the cells were cultured in growth medium for 24 h.

For extracting RNAs, hEPCs were treated with TRIzol reagent (Sigma-Aldrich, MO). For RT-PCR, aliquots of RNA (2 μg each) were subjected to cDNA synthesis with 200 U of Moloney murine leukemia virus reverse transcriptase (Invitrogen) and 0.5 μg of oligo(dT) 15 primer (Promega, Madison, WI). cDNA in 2 μL of the reaction mixture was amplified with 0.5 U of GoTaq DNA polymerase (Promega, Madison, WI) and 10 pmol each of sense and antisense primers, as follows: CXCR2: 5′-CTGCGTACGCTGTTTAAGGC-3′, 5′-GTTGAGGCAGCTGTGAAGGA-3′; GAPDH 5′-TCCATGACAACTTTGGTATCG-3′, 5′-TGTAGCCAAATTCGTTGTCA-3′. The thermal cycle profile was as follows: denaturation at 95 °C for 30 s, annealing at 55 °C for 45 s, and extension at 72 °C for 45 s. Each PCR was performed for 30~35 cycles (depending on the primers used), and PCR products were size-fractionated on 1% ethidium bromide/agarose gels and quantified under UV transillumination.

### Cutaneous wound healing animal model

The 8 weeks old male Sprague–Dawley (SD) rats, average weight 200–250 g (Koatech Inc., Pyeongtaek-city, Republic of Korea), 8~10 weeks old CXCR2 −/− and wild type mice (Jackson Laboratory, Bar Harbor, ME) or BALB/CA-nu/nu mice, average weight 20–24 g (Orient, Seongnam-city, Republic of Korea) were purchased from the corresponding vendors. All animal experiments were performed according to protocols approved by the Institutional Animal Care and Use Committee of Pusan National University Institutional Animal Use and Care Committee. The rats were anesthetized using an intraperitoneal injection of ketamine (50 mg/kg) and xylazine (5 mg/kg), and mice were anesthetized with an intraperitoneal injection of 400 mg/kg Avertin (2,2,2-tribromoethanol; Sigma-Aldrich, St. Louis, MO). The dorsal surface of all animals (rats or mice) was shaved with an electric clipper and an 8-mm or 6-mm biopsy punch was used to create circular, full-thickness excisional wounds on the shaved dorsal skin. Each wound was immediately covered with a dressing film to protect the wound from dryness and self-grooming damage. Wound sites were topically treated with 20 μL HBSS containing the indicated dose of Ac-PGP every day for 12 days. At day 0, 3, 6, 9 and 12 post-wounding, images of wound sites were taken with digital camera. Wound areas in each group were measured using the Image J software (version 1.50i), and percent wound areas calculated in comparison with the original wound at each time point.

### Histological analysis

Animals were sacrificed and tissue samples including the wound area and the surrounding skin were excised. For histological analysis, tissue specimens were formalin-fixed and paraffin-embedded. Three sections measuring 6 μm in thickness were taken from the paraffin-embedded specimens at 150 μm intervals, stained with hematoxylin and eosin (H&E), observed, and photographed with a microscope (Axioimager M2, Carl Zeiss, Heidenheim, Germany). Wound gap, the distance between the advancing edges of epithelial tissue, was measured in three serial sections using Image J software program (version 1.50i).

### Statistical analysis

The results of multiple observations are presented as mean ± SD. For multivariate data analysis, group differences were assessed with one-way or two-way ANOVA, followed by Scheffé's post hoc test.

Additional experimental methods including immunofluorescence staining, *in vivo* tracking of transplanted hEPCs, and 3D immunofluorescence staining with decolorization of wound tissues are included in the supplementary information.

## Additional Information

**How to cite this article**: Kwon, Y. W. *et al*. N-Acetylated Proline-Glycine-Proline Accelerates Cutaneous Wound Healing and Neovascularization by Human Endothelial Progenitor Cells. *Sci. Rep.* **7**, 43057; doi: 10.1038/srep43057 (2017).

**Publisher's note:** Springer Nature remains neutral with regard to jurisdictional claims in published maps and institutional affiliations.

## Supplementary Material

Supplementary Video 1

Supplementary Video 2

Supplementary Video 3

Supplementary Video 4

Supplementary Information
