# Supplementary material for: N-Acetylated Proline-Glycine-Proline Accelerates Cutaneous Wound Healing and Neovascularization by Human Endothelial Progenitor Cells
Source: Sci Rep. 2017 Feb 23;7:43057. doi: 10.1038/srep43057 (PMC5322356; doi:10.1038/srep43057)
Supplement: Supplementary Information [file srep43057-s5.pdf]

Supplementary Information

N-Acetylated Proline-Glycine-Proline Accelerates Cutaneous Wound Healing and Neovascularization by Human Endothelial Progenitor Cells

Yang Woo Kwon, Soon Chul Heo, Tae Wook Lee, Gyu Tae Park, Jung Won Yoon, Il Ho Jang, Seung-Chul Kim[2], Hyun-Chang Ko, Youngjae Ryu, Hyeona Kang, Chang Man Ha, Sang Chul Lee, and Jae Ho Kim

Supplementary Methods

Fluorescence image analysis. Blood vessels were stained with biotinylated-ILB4, rat anti-CD31 (BD) or rabbit anti-α-SMA (Abcam) antibodies. The specimens were incubated with Alexa 488 streptavidin, Alexa 488 or 568 goat anti-rat secondary antibodies (Life Technologies, Carlsbad, CA), followed by washing and mounting in Vectashield medium containing DAPI for visualization of nuclei. The stained sections were visualized by laser scanning confocal microscope (Olympus FluoView FV1000). Blood vessels were counted by identifying ILB4-, CD31- or α-SMA-positive vascular structures per high power field (×200). Four randomly-selected microscopic fields from three serial sections in each tissue block were examined for each wound by two independent observers blinded to the experimental conditions. For *in vivo* tracking of transplanted cells, hEPCs were labeled with the long-lasting Cell Tracker CM-DiI according to the manufacturer's instructions (Invitrogen). The CM-DiI-labeled hEPCs (1×10^6 cells) in HBSS were transplanted into the BALB/CA-nu/nu dermal wound mouse model. hEPCs in 100 μL HBSS or 100 μL HBSS were injected subcutaneously to the respective wound beds at four peripheral sites (25 μL/each site). For quantitative analyses, three sections measuring 6 μm in thickness were taken from the specimens at 150 μm intervals. CM-DiI-labeled cells were counted in four randomly chosen microscopic fields from three serial sections in each tissue block.

Decolorization of dermal wound tissues and 3D immunofluorescence staining. Animals were sequentially perfused with heparin (10 U/ml), 4% PFA, PBS, and 50% CUBIC-1 reagent (25% urea, 25% amino alcohol, 15% Triton X-100)[34]. Square tissue samples including wound area and surrounding skin were excised and immersed in CUBIC-1 reagent

at 37°C for seven days followed by washing on day eight. The transparent tissue samples were immunostained with rat anti-CD31 antibodies for three days, and the stained tissue samples were washed with 0.1% Triton X-100 in PBS for one day, followed by incubation with Alexa 647 goat anti-rat secondary antibodies (Life Technologies, Carlsbad, CA) for three days. The stained samples were washed with 0.1% Triton X-100 in PBS for 1 day and immersed in CUBIC-2 reagent (50% sucrose, 25% urea, 10% amino alcohol, 0.1% Triton X-100) at 37°C for 3-7 days. The stained samples were visualized by light sheet fluorescence microscope (Lightsheet Z.1, Carl Zeiss, Heidenheim, Germany) using the ZEN software (version 2014 SP1). 3D images were generated using the Arivis Vision4D software (version 2.10.4).

Supplementary Figures

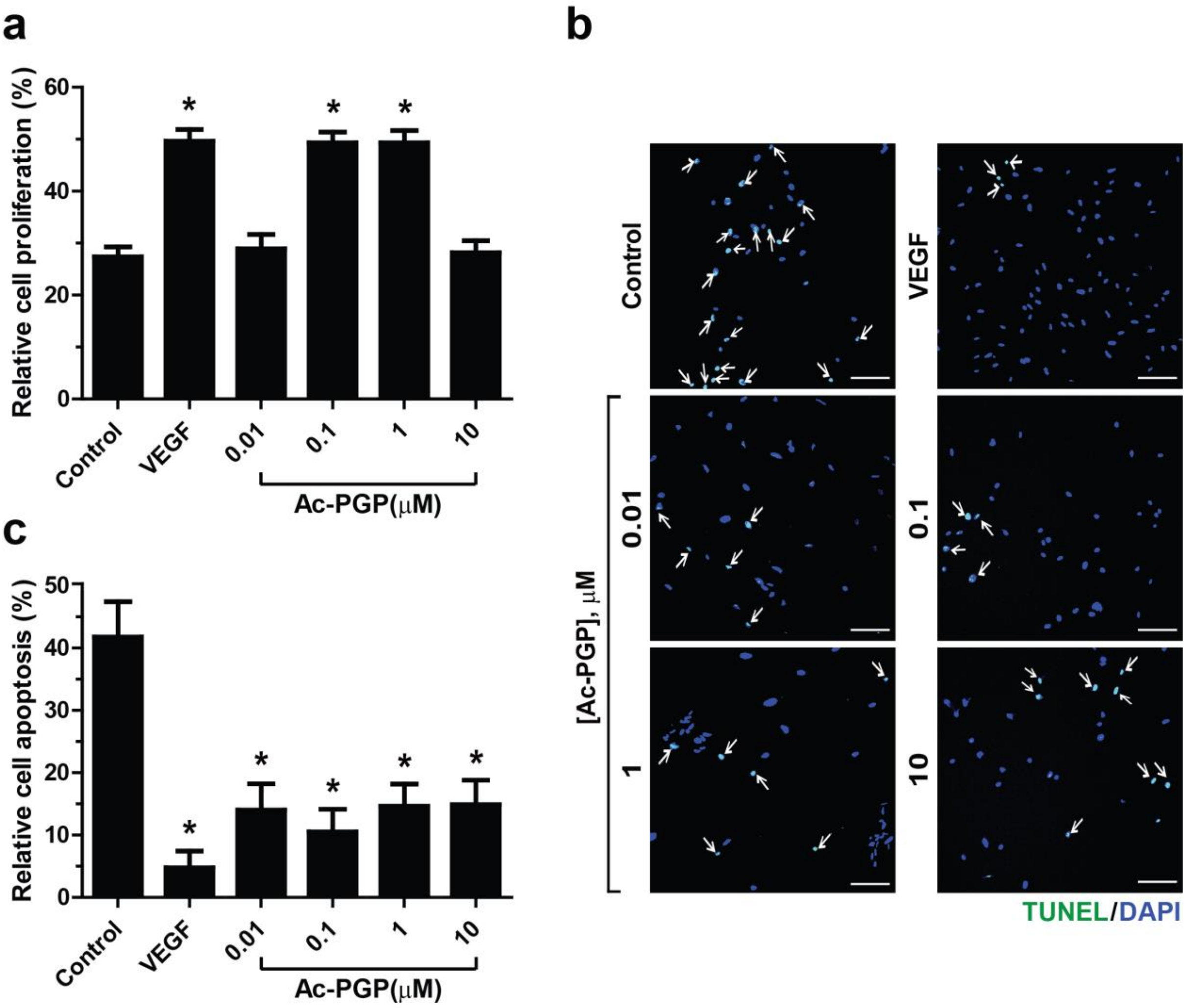

Supplementary Figure S1. Effect of Ac-PGP on proliferation and apoptosis of hEPCs. (**a**) hEPCs proliferation in response to VEGF (10 ng/ml) or various concentrations of Ac-PGP. hEPCs was measured by staining with anti-PCNA. Numbers of PCNA-positive nuclei per field were counted and expressed as the relative percentage of total cells. Data indicate mean ±SD. *p<0.05 versus control (n=6). (**b**) Representative image of hEPC apoptosis in response to VEGF (10ng/ml) or various concentrations of Ac-PGP. (**c**) The apoptotic effect of Ac-PGP on hEPCs, hEPCs was measures by staining TUNEL and DAPI. Number of TUNEL-positive nuclei per field were counted and expressed as the relative percentage of total cells. Data

indicate mean ±SD. *$p<0.05$ versus control (n=6). Bar = 100 µm.

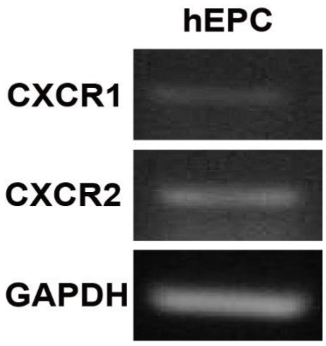

Supplementary Figure S2. CXCR1 and CXCR2 expression in hEPCs. RT-PCR analysis of CXCR1 and CXCR2 expression in hEPCs are shown.

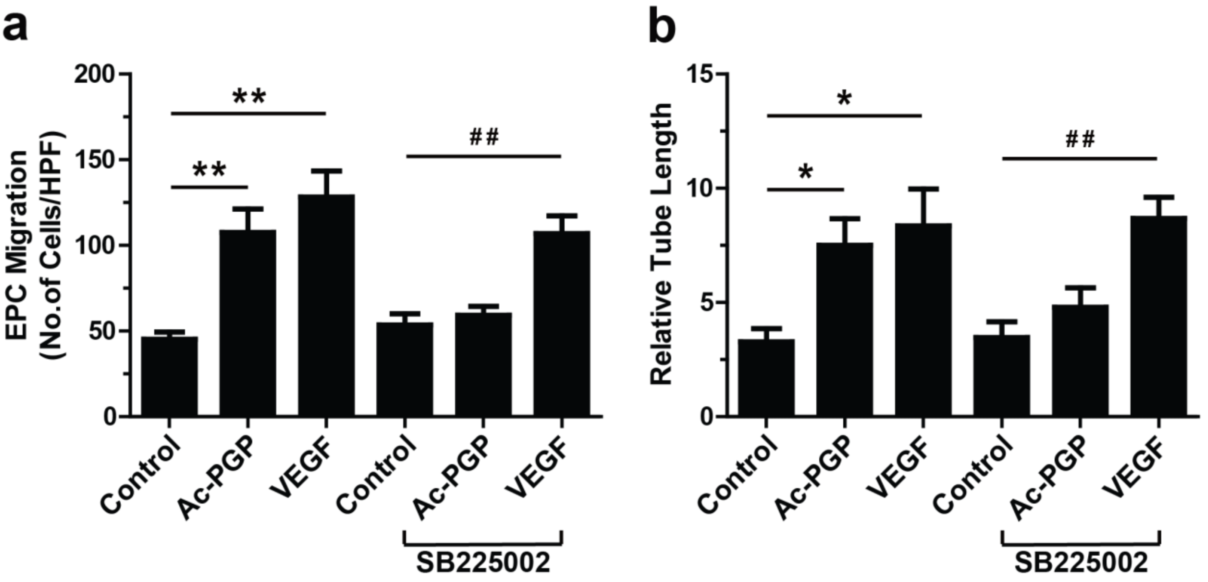

Supplementary Figure S3. Involvement of CXCR2 in the Ac-PGP-induced migration and tube formation of hEPCs. (**a**) hEPCs were treated with vehicle and SB225002, blockade of CXCR2 abrogates Ac-PGP (0.1 µM)-induced migration of hEPCs but not VEGF-induced migration. Data indicate mean ±SD. **p<0.01 versus control, ##p<0.01, SB225002 VEGF versus SB225002 control (n=8) (**b**) Quantitative analysis of tube length by hEPCs in response to Ac-PGP (0.1 µM) or VEGF with or without SB225002. Data indicate mean ±SD. *p<0.5, versus control, ##p<0.01, SB225002 VEGF versus SB225002 control (n=6).

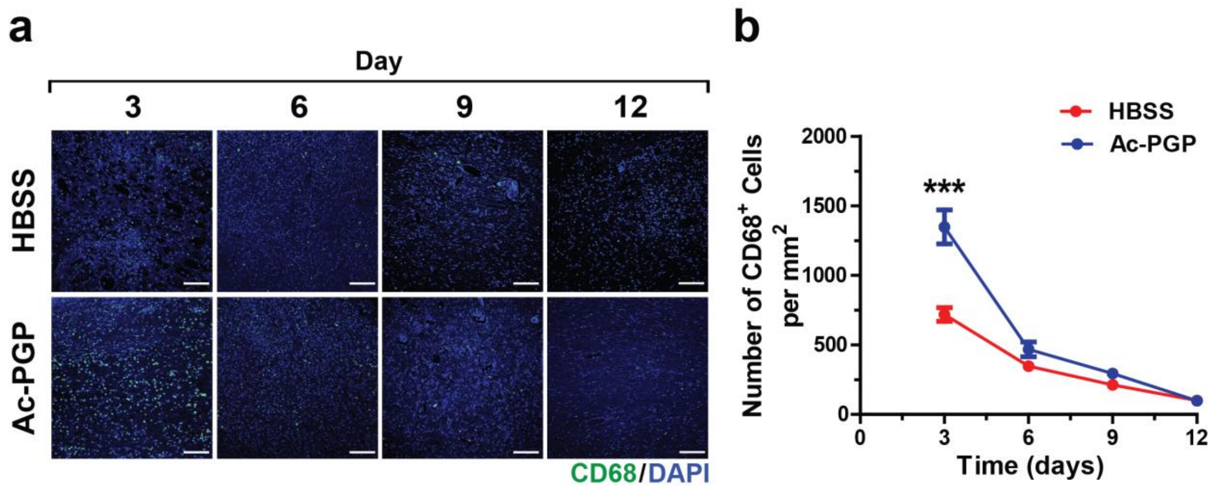

Supplementary Figure S4. Increased infiltration of monocytes/macrophages by Ac-PGP treatment. (**a**) Fluorescence images of CD68 staining (green) in the rat dermal area at indicated days after introducing wound followed by treatment with HBSS or Ac-PGP (0.1 μM) are shown. Nuclei were counterstained with DAPI (blue). Scale bar = 100 μm. (**b**) Quantitative analysis of CD68-positive cells in dermal area by immunohistochemistry is shown. Number of CD68 positive cells was counted at the time point post wound. Data indicate mean ±SD. ***<0.001 versus control (N=8).

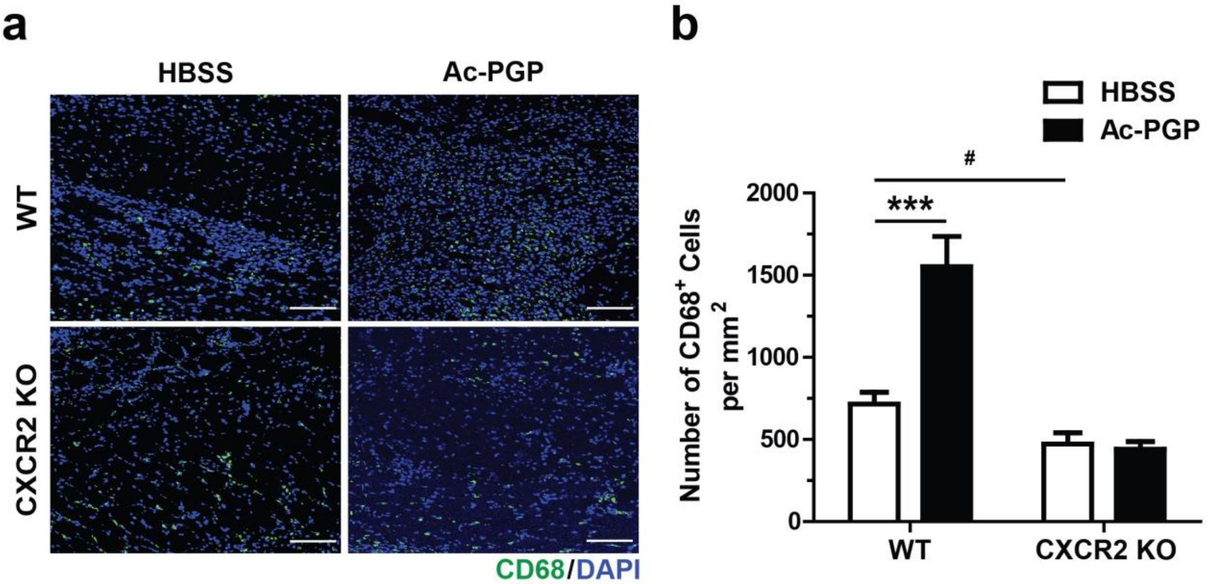

Supplementary Figure S5. Involvement of CXCR2 in Ac-PGP–induced infiltration of monocytes/macrophages in the wound healing model. (**a**) Representative images of mouse skin wound samples at day 3 after immunostaining with anti-CD68 antibody (green) is shown. Nuclei were counterstained with DAPI (blue). Bar = 100 µm. (**b**) Quantitative analysis of CD68-positive cells in the dermal area of day3 skin wound samples is shown. Data indicate mean ±SD. ***p<0.001 WT Ac-PGP versus WT HBSS, #p<0.05 WT HBSS versus CXCR2 KO HBSS (N=8).

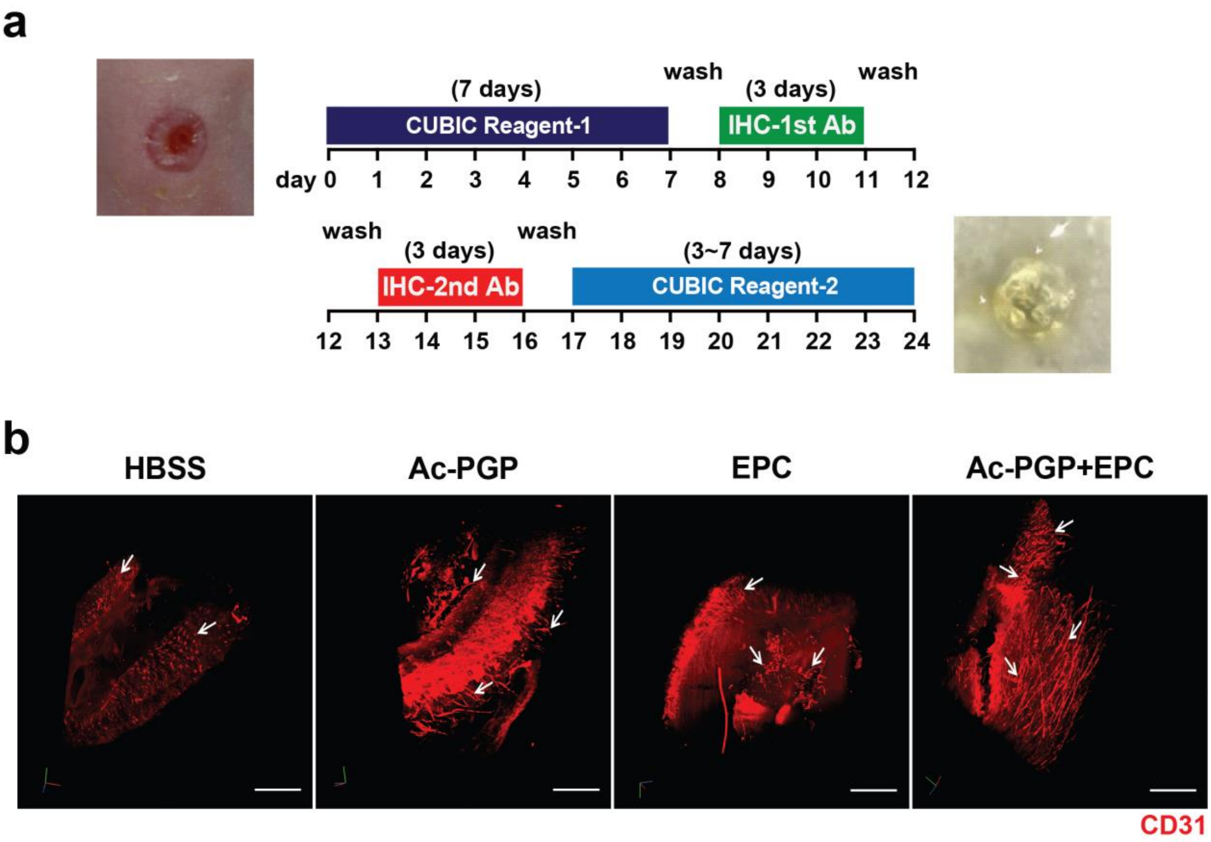

Supplementary Figure S6. Whole mount immunostaining after tissue clearing by CUBIC protocol. (**a**) The tissue clearing procedure and immunohistochemistry (IHC) of wound tissues. A fixed dermal wound (on day 6 after induction of dermal wounds) was treated with CUBIC reagent-1 for seven days, followed by washing with PBS. The wound tissues were stained with anti-CD31. The tissues were then treated with CUBIC reagent-2 for three to seven days to adjust the refractive indices of the tissue and reagent. (**b**) Day 6 mouse skin wound samples were subjected to the whole mount immunohistochemistry with CD31 antibodies after tissue clearing by CUBIC protocol. The reconstituted 3D images corresponding to a quarter of the wound area are shown. White arrows indicate CD31-positive blood vessels. Bar = 1 mm.

Supplementary Video S1-S4. 3D imaging of wound tissues after tissue clearing by CUBIC protocol.

Tissue clearing of wound tissues Cutaneous wound sample from HBSS control (Supplementary Video S1), Ac-PGP treatment (Supplementary Video S2), hEPC injection (Supplementary Video S3), and the combined treatment of Ac-PGP and hEPC injection (Supplementary Video S4) after tissue clearing by CUBIC protocol followed by whole mount immunostaining with anti-CD31 antibody. 3D reconstruction of the area corresponding to a quarter of excised skin is shown.
